# SDS-Assisted Hydrothermal Deposition of NiO Nanoparticles on Carbon Fiber-Reinforced Polymer for Enhanced Supercapacitor Performance

**DOI:** 10.3390/mi17080945

**Published:** 2026-08-07

**Authors:** Claudia Cirillo, Mariagrazia Iuliano, Muhammad Shahzad, Maria Sarno

**Affiliations:** 1 Department of Physics “E.R. Caianiello”, University of Salerno, Via Giovanni Paolo II, 132-84084 Fisciano, Italy; 2 NANO_MATES Research Centre, University of Salerno, Via Giovanni Paolo II, 132-84084 Fisciano, Italy

**Keywords:** carbon fiber-reinforced polymer (CFRP), nickel oxides, hydrothermal synthesis, specific capacitance, supercapacitor

## Abstract

In this study, a CFRP@NiO composite was successfully synthesized via a hydrothermal process using sodium dodecyl sulfate (SDS) as a surfactant. X-ray diffraction (XRD) analysis confirmed the formation of face-centered cubic (FCC) NiO. Morphological characterization revealed that the CFRP exhibited a tube-like structure with an average diameter of approximately 6 μm, uniformly decorated with NiO nanoparticles. The presence of SDS promoted the introduction of hydroxyl (–OH) functional groups onto the CFRP surface, enhancing its surface functionality and facilitating the homogeneous deposition of NiO nanoparticles. This surface modification also contributed to the improved thermal stability of the CFRP@NiO composite. Electrochemical measurements demonstrated a remarkable enhancement in capacitive performance. Cyclic voltammetry (CV) results showed that the specific capacitance increased from 10 to 135 F g^−1^ at a scan rate of 5 mV s^−1^ after NiO incorporation. Furthermore, galvanostatic charge–discharge (GCD) analysis revealed significantly extended charge–discharge times, confirming the superior energy storage capability of the CFRP@NiO composite compared with pristine CFRP.

## 1. Introduction

The rapid global transition toward sustainable and renewable energy has significantly increased the demand for efficient, reliable, and environmentally friendly energy-storage systems. Among the available technologies, supercapacitors, also known as electrochemical capacitors, have attracted considerable attention because of their high power density, long cycle life, rapid charge–discharge capability, and excellent operational stability over a wide temperature range [1,2,3,4]. These characteristics make them suitable for applications in portable electronics, electric vehicles, and smart-grid systems [5,6,7]. Nevertheless, their relatively low energy density compared with conventional batteries, narrow operating-voltage window, and limited long-term electrochemical stability remain major obstacles to widespread implementation [8,9,10]. Addressing these limitations requires advanced electrode materials that combine high electrochemical activity with structural stability and mechanical integrity [11,12].

Conducting polymers, including polyaniline (PANI), polypyrrole (PPy), and poly (3,4-ethylenedioxythiophene) (PEDOT), have been extensively investigated as supercapacitor electrode materials because their reversible Faradaic redox reactions provide high pseudocapacitance [13,14,15,16,17]. Compared with conventional carbon-based electric double-layer capacitors, these polymers can deliver higher energy density while retaining lightweight and flexible characteristics, which are attractive for wearable and flexible electronic devices [18,19]. However, their practical use is constrained by poor cycling stability, substantial volume expansion and contraction during repeated redox reactions, and limited mechanical durability, which can cause rapid capacitance degradation [20]. Hybrid composite electrodes have therefore been developed to combine electrochemically active components with conductive and mechanically robust supports.

Carbon fiber-reinforced polymers (CFRPs) are promising multifunctional supports because of their high mechanical strength, low density, thermal stability, and intrinsic electrical conductivity [21,22,23,24]. They can serve as lightweight, mechanically robust current collectors or structural substrates for electroactive materials in structural supercapacitors [25]. Pristine CFRPs, however, generally exhibit low capacitance because their surfaces provide few electrochemically active sites and do not support substantial Faradaic reactions. The integration of CFRPs with nanostructured metal oxides can preserve their mechanical advantages while introducing pseudocapacitive activity, increasing the accessible surface area, and improving ion-transport pathways [26].

Recent studies have demonstrated that surface engineering can substantially improve the electrochemical response of carbon fiber-based structural electrodes. Graphene-aerogel-modified carbon fibers reached a specific capacitance of 92.5 F g^−1^ with 98% capacitance retention after 10,000 charge–discharge cycles [21]. Graphene-nanoplatelet-coated carbon fiber electrodes and polyaniline-coated carbon fabrics have also shown improved capacitive performance under binder-free or structural configurations [27,28,29]. Furthermore, thermal and chemical treatments of reclaimed carbon fibers, combined with Cu_2_O/CuO nanoparticles and oxygen-containing functional groups, increased the specific capacitance to 122.2 F g^−1^ while maintaining stability over 30,000 cycles [21,27,28,29]. These findings show that surface functionalization and nanostructured coatings are effective routes for improving carbon fiber-based electrodes.

A persistent limitation is the weak interface between nanomaterials and the chemically inert, relatively smooth CFRP surface. Poor wettability and insufficient interfacial adhesion can impair nanoparticle anchoring, mechanical integrity, and charge transfer. Surfactant-assisted surface modification provides a practical strategy for overcoming these limitations. Cationic surfactants such as cetyltrimethylammonium bromide (CTAB) can enhance electrostatic interactions with negatively charged surfaces, whereas nonionic surfactants such as Triton X-100 and Tween 80 mainly provide steric stabilization. Sodium dodecyl sulfate (SDS) is particularly attractive because of its anionic character, low cost, and ability to regulate nanoparticle nucleation and growth during hydrothermal synthesis. As a micellar template and electrostatic stabilizer, SDS can reduce particle agglomeration and promote uniformly dispersed, fine-grained nanostructures [30,31]. It can also increase carbon fiber wettability and mitigate cathodic degradation under galvanic coupling conditions [32]. In NiO systems, SDS has been reported to influence crystallinity, morphology, electrochemical activity, charge-transfer kinetics, and capacitive performance, making it an attractive surfactant for the fabrication of high-performance composite electrodes [33,34].

NiO is a promising pseudocapacitive material because of its high theoretical specific capacitance, redox activity, and cycling stability. Although NiO/carbon composites have been widely investigated, most reported architectures use carbon cloth, graphene, carbon nanotubes, or activated carbon as the supporting phase [35,36,37]. The present work differs by employing reclaimed CFRP waste as a mechanically robust and sustainable substrate. SDS is used not only to assist hydrothermal nanoparticle formation but also to improve CFRP surface wettability and promote the homogeneous nucleation, anchoring, and interfacial interaction of NiO nanoparticles. Accordingly, a CFRP@NiO composite was synthesized by an SDS-assisted hydrothermal route, and its structural, morphological, thermal, and electrochemical properties were systematically evaluated to determine its potential as an electrode material for lightweight structural supercapacitors.

## 2. Materials and Methods

### 2.1. Materials

Reclaimed carbon fiber-reinforced polymer (CFRP) (Batch E) was supplied by AEROSOFT (Naples, Italy). Nickel (II) chloride hexahydrate (NiCl_2_·6H_2_O), sodium dodecyl sulfate (SDS, NaC_12_H_25_SO_4_), and urea (CO(N_2_) _22_) were used as precursor materials for the synthesis of the CFRP@NiO composite. All chemicals were purchased from Sigma-Aldrich (St. Louis, MO, USA) and Merck KGaA (Darmstadt, Germany) with a minimum purity of 98% and were used without further purification.

### 2.2. Preparation of CFRP

The reclaimed CFRP (Batch E) was cleaned to remove residual impurities before surface modification. Initially, 1 g of CFRP was dispersed in 5 mL of N,N-dimethylformamide (DMF), ultrasonicated for 5 min, and subsequently centrifuged at 7500 rpm for 30 min. The supernatant was discarded, and the recovered solid was collected. The cleaning procedure was repeated sequentially using acetone and distilled water under the same conditions. Finally, the washed sample was rinsed with ethanol and dried in an oven at 60 °C for 24 h.

### 2.3. Synthesis of the CFRP@NiO Composite

The CFRP@NiO composite was synthesized using a hydrothermal method. Briefly, 0.5 g of pretreated CFRP, 0.5 g of NiCl_2_·6H_2_O, and 20 g of urea were dispersed in 25 mL of distilled water and magnetically stirred for 3 h to obtain a homogeneous precursor solution. Subsequently, 0.10 g of SDS was added to the precursor solution under continuous magnetic stirring until complete dissolution. The resulting suspension was transferred into a Teflon-lined stainless-steel autoclave and maintained at 80 °C for 4 h under hydrothermal conditions. After the reaction was completed, 60 mL of ethanol was added, and the suspension was centrifuged at 7000 rpm for 30 min. The precipitate was collected and washed several times with distilled water and ethanol to remove residual reactants and by-products. The purified product was dried in an oven at 60 °C for 24 h and subsequently calcined at 300 °C for 6 h to obtain the final CFRP@NiO composite.

### 2.4. Characterization

The crystalline structure of the synthesized materials was characterized by X-ray diffraction (XRD) using a Bruker D2 diffractometer (Bruker AXS, Karlsruhe, Germany) with Cu Kα radiation (λ = 1.5406 Å). The surface morphology and elemental composition were investigated using scanning electron microscopy (SEM, TESCAN VEGA LMH 230 V) ((TESCAN Brno, s.r.o., Brno, Czech Republic)) equipped with an energy-dispersive X-ray spectroscopy (EDS/EDX) detector (AMETEK, Berwyn, PA, UAS). The surface functional groups were identified by Fourier-transform infrared (FTIR) spectroscopy using a Thermo Scientific Nicolet iS50 spectrometer ((Thermo Fisher Scientific, Waltham, MA, USA)), while the thermal stability of the samples was evaluated by thermogravimetric analysis (TGA). Electrochemical measurements were carried out using an Autolab PGSTAT302N potentiostat/galvanostat workstation (Metrohm, Herisau, Switzerland).

### 2.5. Electrochemical Measurements

The electrochemical performance of the CFRP@NiO electrode was evaluated using a conventional three-electrode configuration. The CFRP@NiO composite served as the working electrode, a platinum plate was employed as the counter electrode, and an Ag/AgCl electrode was used as the reference electrode. In particular, the working electrode was prepared by dispersing 80 wt% CFRP@NiO, 10 wt% acetylene black, and 10 wt% polyvinylidene fluoride (PVDF) in an appropriate amount of N-methyl-2-pyrrolidone (NMP) to form a homogeneous slurry. The resulting ink was uniformly coated onto nickel foam (1 × 1 cm^2^) and dried under vacuum at 80 °C for 12 h to remove the solvent. Subsequently, the electrode was pressed under pressure of 20 MPa to improve the contact between the active material and the current collector. The mass loading of the active material was approximately 3.0 mg/cm^2^. All electrochemical measurements were performed in a 1 M KOH aqueous electrolyte. Cyclic voltammetry (CV) measurements were conducted using an Autolab PGSTAT302N potentiostat ((Metrohm Autolab B.V., Utrecht, The Netherlands)) over scan rates ranging from 5 to 50 mV s^−1^ to evaluate the capacitive behavior of the prepared electrode. Electrochemical impedance spectroscopy (EIS) measurements were also carried out in the same electrolyte to investigate the charge-transfer characteristics and electrochemical resistance of the composite electrode.

## 3. Results and Discussion

### 3.1. Crystal Structure Analysis

**Figure 1:**
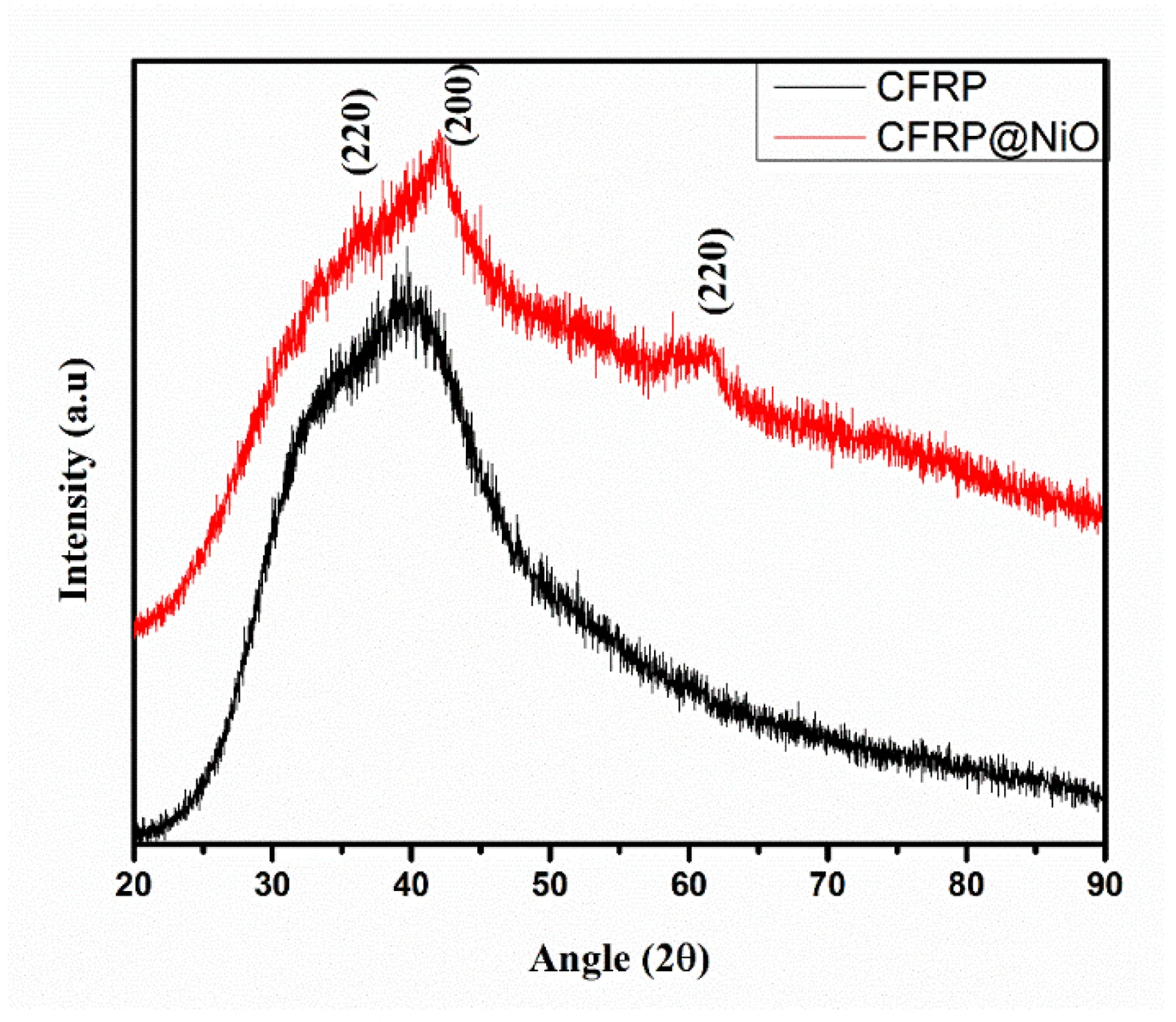
XRD patterns of pristine CFRP and the CFRP@NiO composite.

The X-ray diffraction (XRD) patterns of pristine CFRP and the hydrothermally synthesized CFRP@NiO composite are presented in Figure 1. The diffraction pattern of pristine CFRP exhibits a broad peak, indicating its predominantly semi-crystalline structure, which arises from the coexistence of amorphous polymer regions and partially ordered carbon fibers. Following hydrothermal treatment in the presence of SDS, several distinct diffraction peaks appeared at 2θ values of 36.31°, 42.10°, and 61.64°, corresponding to the (111), (200), and (220) crystallographic planes of cubic NiO [38], in good agreement with the standard JCPDS card No. 47-1049. These reflections are characteristic of the face-centered cubic (FCC) phase of NiO, confirming the successful formation and deposition of crystalline NiO nanoparticles on the CFRP surface. The absence of additional impurity peaks further suggests the high purity of the synthesized composite.

### 3.2. Morphological and Elemental Analysis

**Figure 2:**
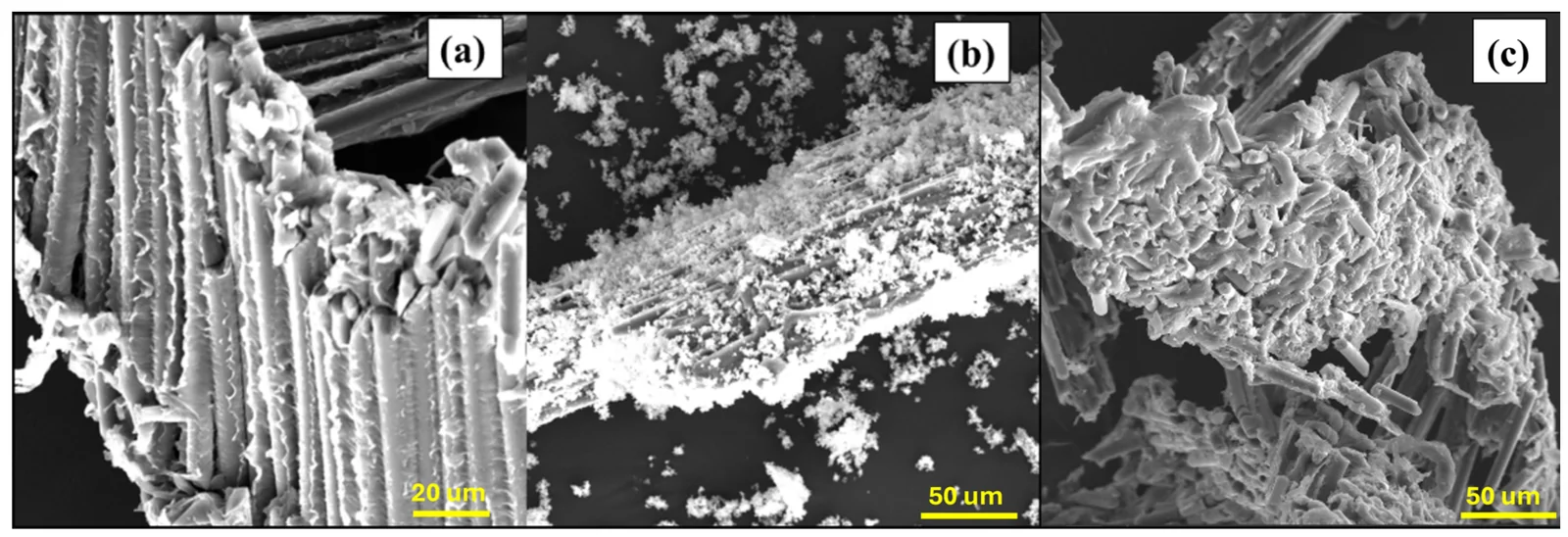
SEM images of pristine CFRP (**a**), CFRP@NiO composite (**b**) and CFRP@NiO composite without SDS (**c**).

**Figure 3:**
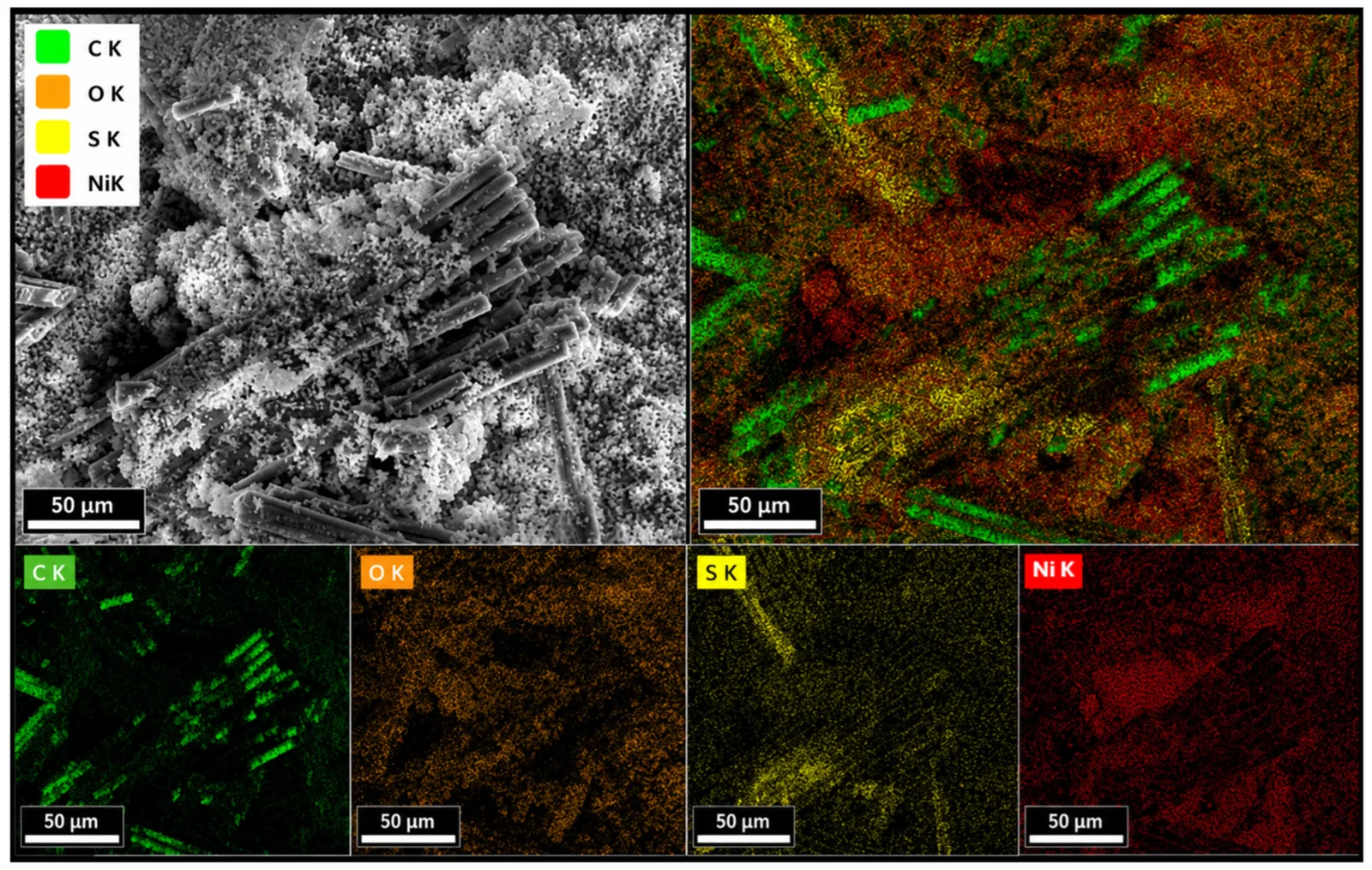
SEM image and EDS elemental mapping of the CFRP@NiO composite showing the distribution of C, O, S, and Ni.

The surface morphologies of CFRP, CFRP@NiO and CFRP@NiO without SDS are shown in Figure 2. The pristine CFRP consists of smooth tubular fibers with an average diameter of approximately 6 μm (Figure 2a). After hydrothermal synthesis, numerous NiO nanoparticles were uniformly deposited on the fiber surface without causing noticeable changes to the original fibrous morphology (Figure 2b). Figure 2c shows the morphology of the CFRP@NiO sample synthesized in the absence of SDS. Compared with the SDS-assisted sample, only a limited number of NiO nanoparticles are observed on the carbon fiber surface, while the original smooth morphology of the CFRP is largely preserved. The reduced particle density indicates that, in the absence of SDS, the hydrothermal process is less effective in promoting the nucleation and anchoring of NiO nanoparticles onto the carbon fibers. These observations highlight the crucial role of SDS in improving the wettability of the CFRP surface, facilitating homogeneous nucleation, and promoting the uniform growth and adhesion of NiO nanoparticles during hydrothermal synthesis. The roughened surface generated by the NiO coating is expected to provide additional electrochemically active sites and facilitate electrolyte penetration. The elemental distribution of the CFRP@NiO composite was investigated by energy-dispersive X-ray spectroscopy (EDS) elemental mapping, as shown in Figure 3. The elemental maps reveal a homogeneous distribution of carbon (C), oxygen (O), sulfur (S), and nickel (Ni) throughout the composite. Carbon is primarily associated with the CFRP substrate, whereas the oxygen and nickel signals overlap, confirming the successful formation and uniform deposition of NiO nanoparticles on the CFRP surface. The presence of sulfur originates from the residual SDS used during hydrothermal synthesis, indicating its contribution to the surface functionalization process. Quantitative EDS analysis revealed elemental contents of 51.10 wt.% carbon, 29.77 wt.% oxygen, and 16.98 wt.% nickel, further confirming the successful incorporation of NiO into the CFRP matrix. The homogeneous elemental distribution demonstrates the effective interaction between the functionalized CFRP surface and the deposited NiO nanoparticles.

### 3.3. Functional Group Analysis

**Figure 4:**
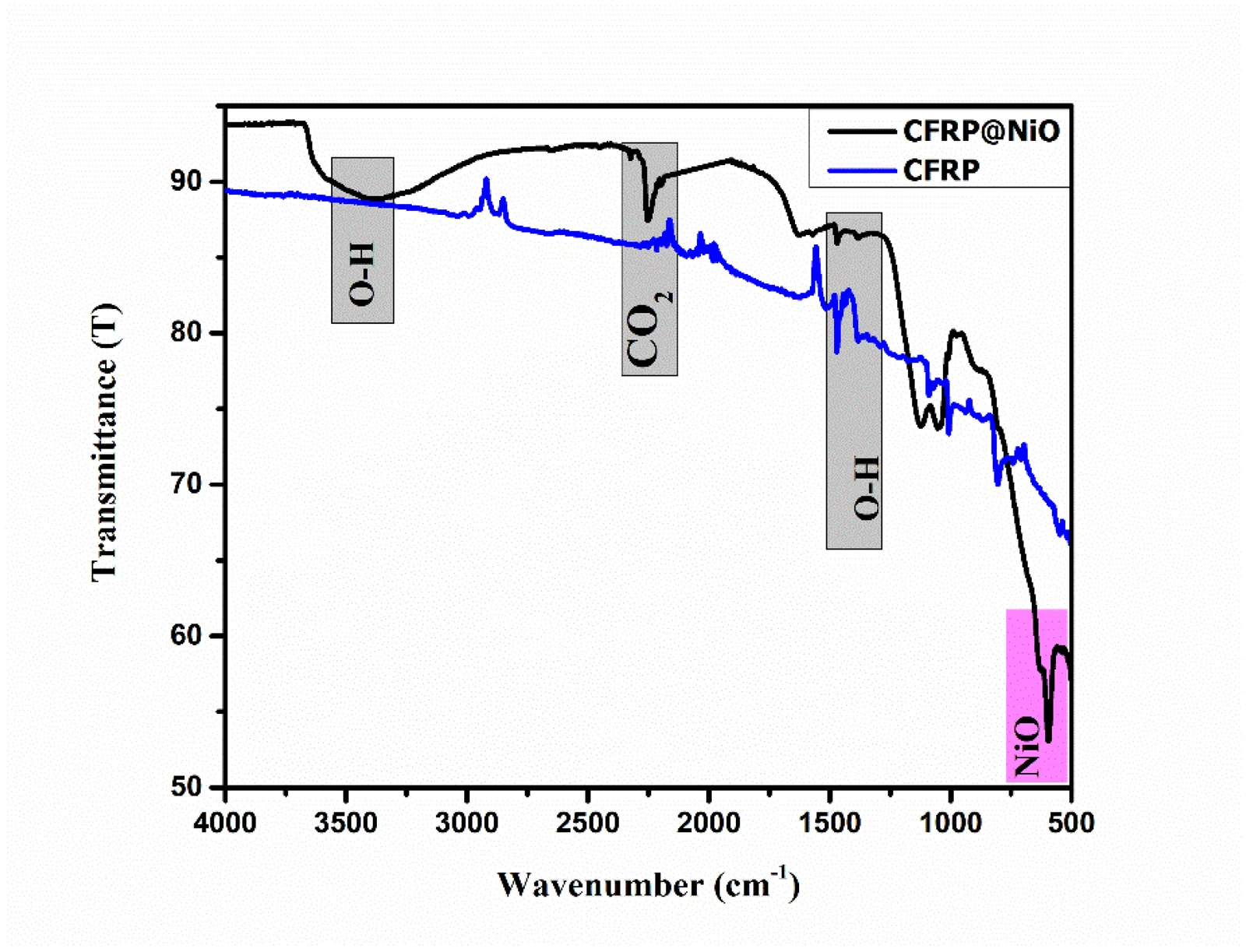
FTIR spectra of pristine CFRP (blue line) and the CFRP@NiO composite (black line).

The FTIR spectra of pristine CFRP and CFRP@NiO are presented in Figure 4. The pristine CFRP exhibits only a weak absorption band at approximately 1385 cm^−1^, indicating a limited number of oxygen-containing functional groups on the fiber surface. Following hydrothermal treatment, the CFRP@NiO composite displays a broad absorption band centered at 3387 cm^−1^ corresponding to O–H stretching vibrations, together with a peak at 2252 cm^−1^ associated with adsorbed CO_2_. A characteristic absorption band observed at 588 cm^−1^ is assigned to Ni–O stretching vibrations, confirming the formation of nickel oxide [39]. The increased intensity of the O–H band indicates a higher concentration of oxygen-containing functional groups after hydrothermal treatment. This observation is consistent with the role of SDS reported in the literature, where the surfactant enhances the surface wettability of carbon materials and facilitates the nucleation and anchoring of NiO nanoparticles.

### 3.4. Thermal Analysis

**Figure 5:**
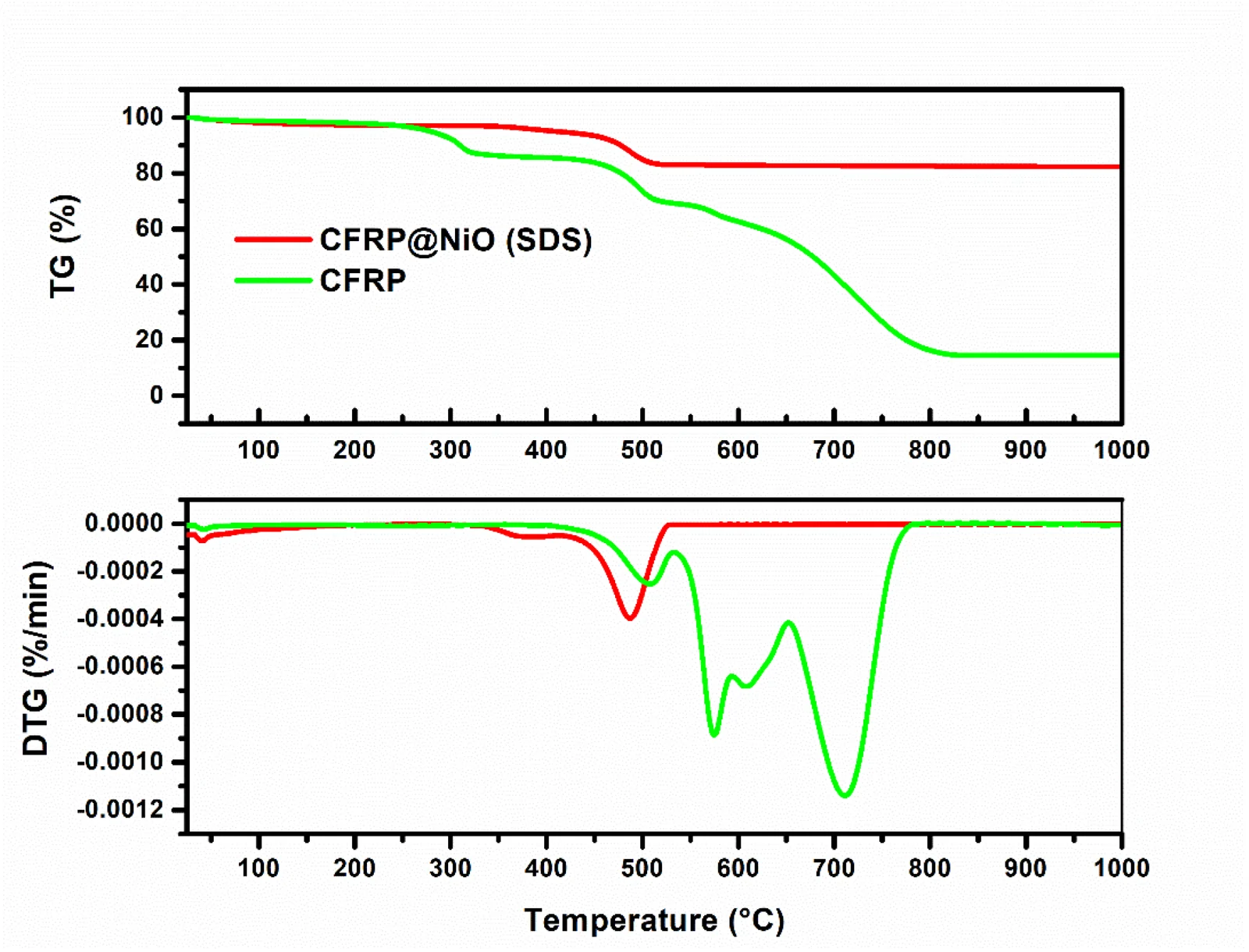
TGA and DTG curves of pristine CFRP and the CFRP@NiO composite.

The thermal stability of the prepared materials was evaluated by thermogravimetric analysis (TGA), as shown in Figure 5. Pristine CFRP undergoes three successive weight-loss stages. The initial weight loss (approximately 2%) is attributed to the evaporation of physically adsorbed moisture. The second stage corresponds to degradation of the polymer matrix accompanied by the formation of volatile products and carbonaceous charts. The final stage is associated with combustion of the remaining polymer residues and degradation of the carbon fiber structure. Compared with pristine CFRP, the CFRP@NiO composite exhibits enhanced thermal stability throughout the investigated temperature range. This improvement can be attributed to the strong interaction between NiO nanoparticles and the functionalized CFRP surface generated during hydrothermal treatment in the presence of SDS. Enhanced interfacial interaction effectively delays thermal degradation and improves the thermal resistance of the composite [40,41].

### 3.5. Electrochemical Performance

**Figure 6:**
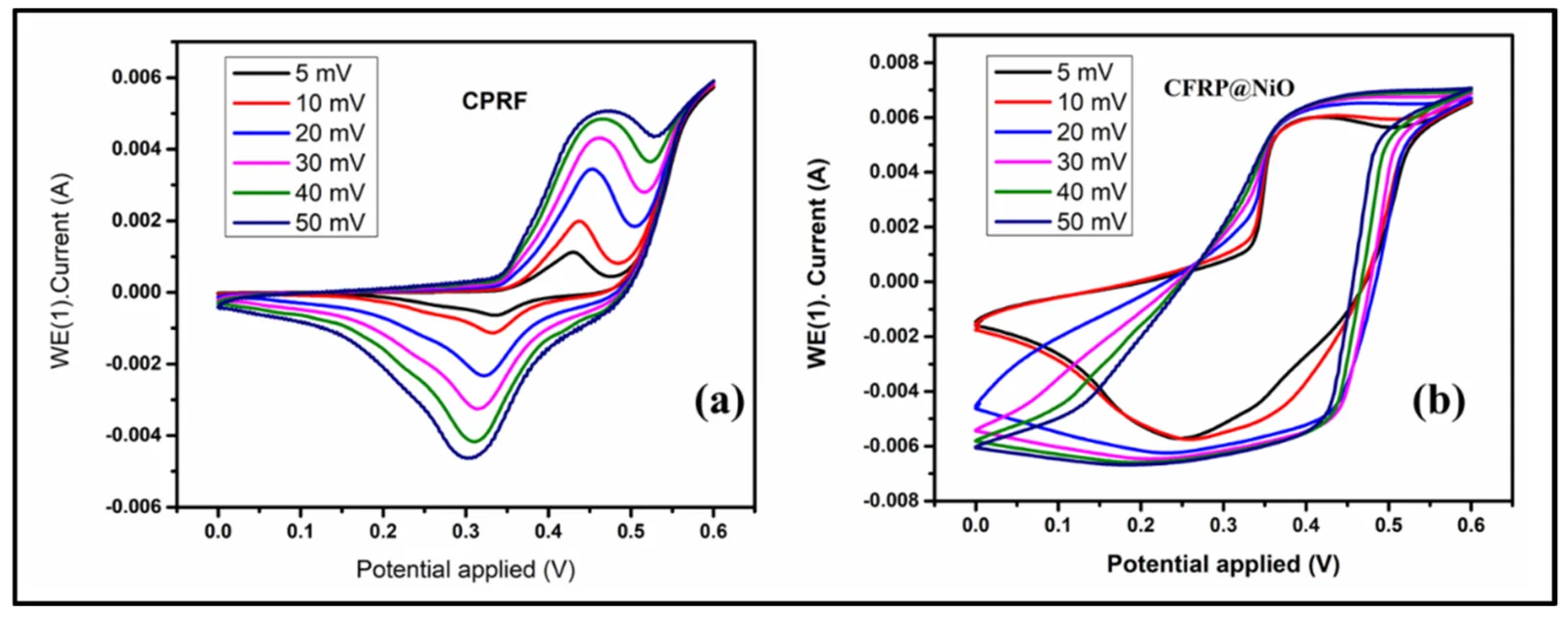
Cyclic voltammograms of (**a**) pristine CFRP and (**b**) CFRP@NiO.

**Figure 7:**
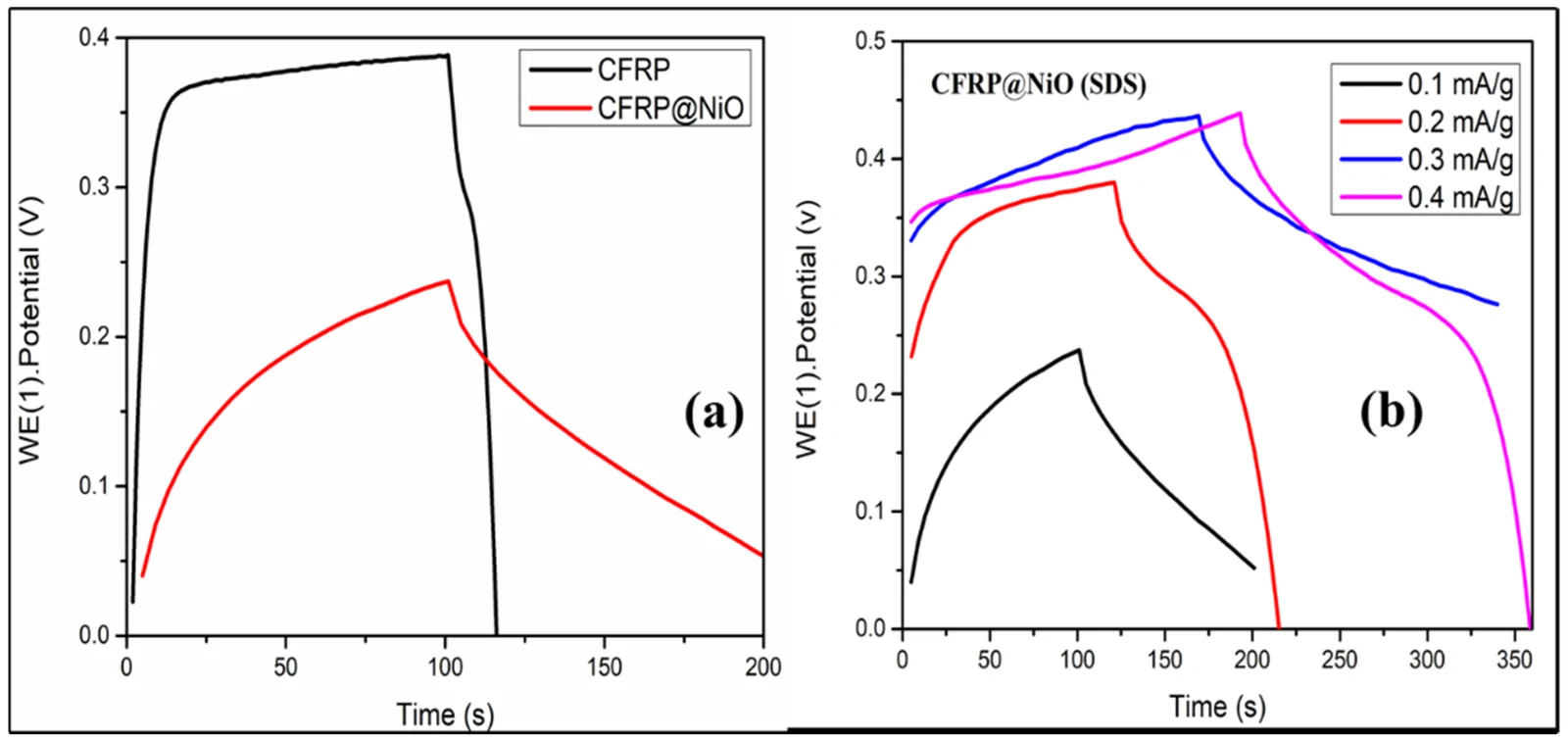
Galvanostatic charge–discharge curves of (**a**) CFRP and CFRP@NiO and (**b**) CFRP@NiO at different current densities.

The cyclic voltammograms (CV) of pristine CFRP and the CFRP@NiO composite recorded in 1 M KOH electrolyte are presented in Figure 6. Pristine CFRP exhibits weak redox peaks between 0.33 and 0.42 V, whereas the CFRP@NiO composite displays enhanced redox activity with slightly shifted oxidation and reduction peaks. These potential shifts may be attributed to changes in charge-transfer kinetics, surface polarization, and electrode/electrolyte interfacial interactions following NiO deposition. A significant enhancement in electrochemical performance was achieved after the incorporation of NiO nanoparticles. The CFRP@NiO electrode exhibited a maximum specific capacitance of 135 F g^−1^, representing an approximately 13-fold increase over pristine CFRP (10 F g^−1^). This remarkable improvement is attributed to the pseudocapacitive behavior of NiO together with the enlarged electrochemically active surface area provided by the uniformly deposited nanoparticles. In addition, the porous NiO coating facilitates electrolyte penetration and enhances charge-transfer kinetics at the electrode/electrolyte interface, resulting in improved capacitive performance [42,43]. The galvanostatic charge–discharge (GCD) curves recorded at different current densities are shown in Figure 7. Compared with pristine CFRP, the CFRP@NiO electrode exhibits significantly longer charge and discharge times, confirming its superior charge-storage capability. The nearly symmetric shape of the GCD profiles demonstrates the good reversibility of the charge–discharge process and the highly capacitive behavior of the composite electrode. The Coulombic efficiency (CE), calculated as the ratio between the discharge and charge times at the same current density [44,45], was approximately 97%, indicating efficient charge storage with limited irreversible charge losses during the electrochemical process. These results further confirm that the hydrothermally grown NiO nanosheets effectively enhance the electrochemical activity of the carbon fiber felt, making the CFRP@NiO composite a promising electrode material for high-performance supercapacitor applications.

### 3.6. Electrochemical Impedance Spectroscopy (EIS)

**Figure 8:**
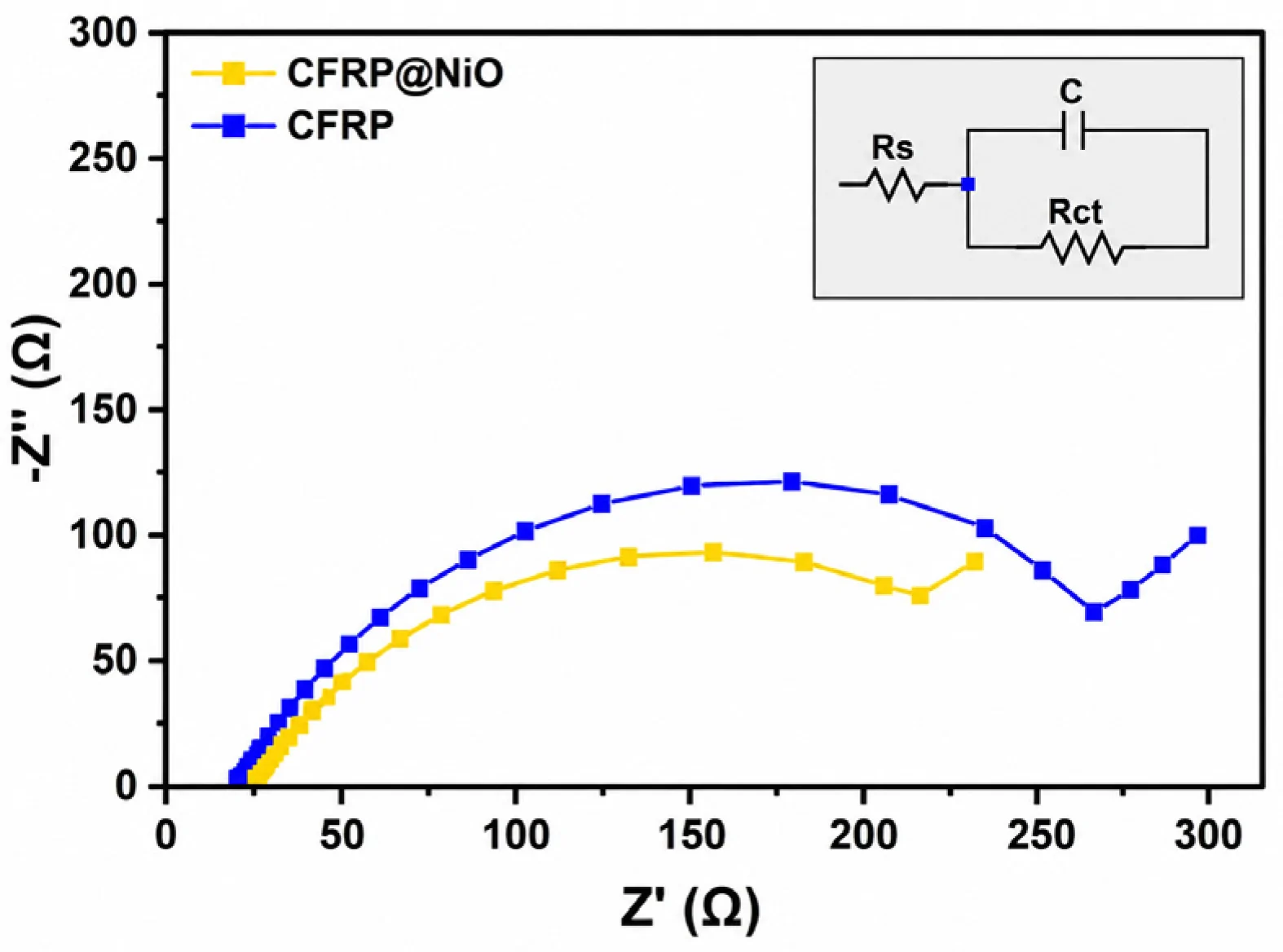
Electrochemical impedance spectroscopy (EIS) Nyquist plots of pristine CFRP and CFRP@NiO electrodes in 1 M KOH electrolyte.

Figure 8 presents the Nyquist plots of the pristine CFRP and the CFRP@NiO electrodes, together with the corresponding Randles equivalent circuit used to describe the electrochemical behavior. Both electrodes exhibit a similar impedance profile consisting of a depressed semicircle in the high-frequency region followed by a low-frequency diffusion region. The high-frequency semicircle is associated with the charge-transfer resistance (Rct) at the electrode/electrolyte interface, while the low-frequency region reflects the ion diffusion process within the porous electrode structure. Compared with pristine CFRP, the CFRP@NiO electrode exhibits a smaller semicircle diameter, indicating a lower charge-transfer resistance. This behavior demonstrates that the hydrothermally deposited NiO nanoparticles improve the electrical contact between the active material and the CFRP substrate, facilitating electron transport and accelerating the Faradaic redox reactions. The lower charge-transfer resistance observed for the CFRP@NiO electrode is attributed to the homogeneous distribution of NiO nanoparticles. Such morphology is consistent with the beneficial effect of SDS reported in previous studies [46,47], where the surfactant improves nanoparticle dispersion and interfacial contact.

### 3.7. Cycling Stability

**Figure 9:**
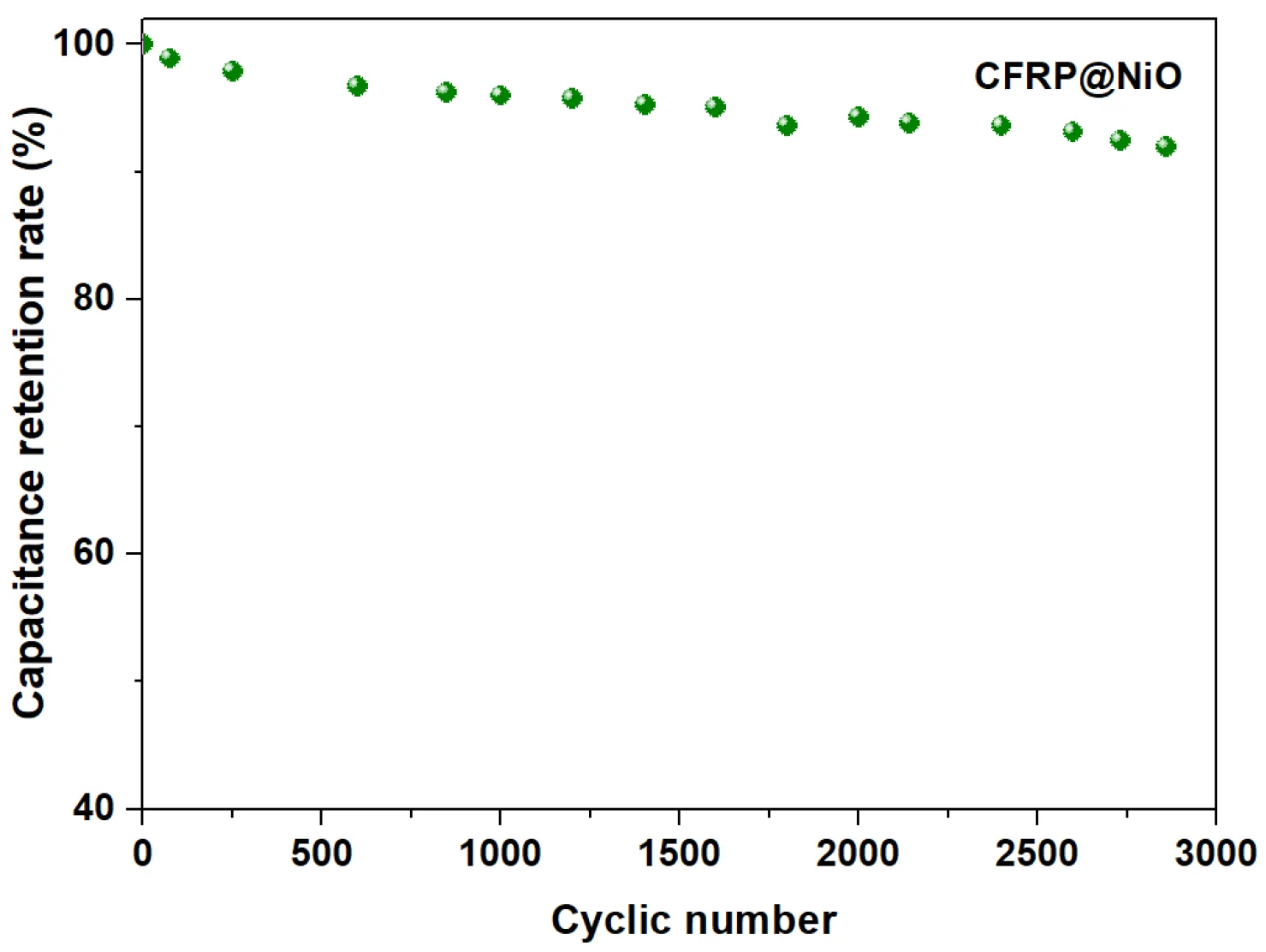
Cycling performance and capacitance retention for CFRP@NiO electrode in 1 M of KOH solution.

To evaluate the long-term electrochemical stability of the CFRP@NiO electrode, galvanostatic charge–discharge cycling tests were performed in 1 M KOH electrolyte for approximately 3000 charge–discharge cycles (Figure 9). The specific capacitance gradually decreased during the initial cycles and then stabilized, indicating the structural integrity and a robust electrode/electrolyte interface in the CFRP@NiO composite. After approximately 3000 cycles, the electrode retained 91% of its initial capacitance, confirming its good cycling durability. The high capacitance retention can be attributed to the homogeneous distribution of NiO nanoparticles on the functionalized CFRP surface, which promotes efficient electron transport and facilitates electrolyte ion diffusion while minimizing structural degradation during repeated charge–discharge processes. These results demonstrate that the SDS-assisted hydrothermal deposition of NiO onto reclaimed CFRP provides a stable electrode architecture with good electrochemical reversibility, making the CFRP@NiO composite a promising candidate for long-life supercapacitor applications.

### 3.8. Performance Comparison with Reported NiO-Based Composite Electrodes

Table 1 compares the electrochemical performance of the present CFRP@NiO electrode with representative NiO-based electrodes reported in the literature. Although the specific capacitance of the CFRP@NiO electrode (135 F g^−1^ at 5 mV s^−1^) is lower than that of several highly engineered NiO/carbon nanocomposites, it is higher than that of pristine NiO nanoparticles synthesized via a green route (85.31 F g^−1^) [48]. More importantly, the CFRP@NiO electrode exhibits excellent cycling stability, retaining 96.1% of its initial capacitance after 1000 charge–discharge cycles. The comparatively lower capacitance can be attributed to the use of reclaimed CFRP as the supporting substrate, which provides lower specific surface area than graphene- or CNT-based architectures but offers superior mechanical robustness and sustainability.

## 4. Conclusions

The CFRP@NiO composite was successfully fabricated through a hydrothermal route in the presence of SDS, which effectively functionalized the CFRP surface and promoted the uniform deposition of NiO nanoparticles. Structural, morphological, and spectroscopic analyses confirmed the successful formation of crystalline NiO and its homogeneous distribution over the CFRP substrate, while the surface functionalization contributed to enhanced thermal stability. Electrochemical measurements revealed a remarkable improvement in capacitive performance, with the specific capacitance increasing from 10 to 135 F g^−1^ after NiO incorporation. In addition, the prolonged charge–discharge response observed for the CFRP@NiO electrode demonstrated its enhanced charge-storage capability. These findings highlight the effectiveness of SDS-assisted hydrothermal deposition as a simple and efficient strategy for producing high-performance CFRP-based electrodes, making the developed composite a promising candidate for lightweight structural supercapacitors and next-generation energy-storage devices.

## Figures and Tables

**Table 1:** Comparison of the electrochemical performance of the present CFRP@NiO electrode with representative NiO-based composite electrodes reported in the literature.

Composite Electrodes	Fabrication Method	Specific Capacitance	Cycling Stability	Ref.
Sucrose-derived carbon-coated NiO	Colloidal method	473 F g^−1^ at 5 mV s^−1^	NA	[49]
Porous NiO/C nanocomposite	Calcination	644 F g^−1^ at 2 mV s^−1^	12% loss after 1000 cycles	[50]
Multilayered NiO/CNT	Hydrothermal	713.9 F g^−1^ at 2 mV s^−1^	11.8% loss after 3000 cycles	[51]
NiO nanoparticles	Green synthesis	85.31 F g^−1^ at 2 mV s^−1^	NA	[48]
Nanoporous NiO film	Electrochemical deposition	797 F g^−1^ at 5 mV s^−1^	5.2% loss after 500 cycles	[52]
**CFRP@NiO**	**SDS-assisted hydrothermal synthesis**	**135 F g^−1^ at 5 mV s^−1^**	**96.1% capacitance retention after 1000 cycles**	**This work**

## Data Availability

The data presented in this study are available on request from the corresponding author.
